# Correction to “Rib‐Origin Ewing Sarcoma With Cranial Nerve Involvement From Orbital Metastasis: A Rare Case Report”

**DOI:** 10.1002/ccr3.73410

**Published:** 2026-08-30

M. N. A. Khan , K. Ghufran , M. Tausif , et al., “Rib‐Origin Ewing Sarcoma With Cranial Nerve Involvement From Orbital Metastasis: A Rare Case Report,” Clinical Case Reports 14, no. 3 (2026): e72309, 10.1002/ccr3.72309.42016629 PMC13093329

The color descriptions for the red and blue arrows were inadvertently reversed in the second paragraph of Section 2.3 and in the caption of Figure 1.

**FIGURE 1:**
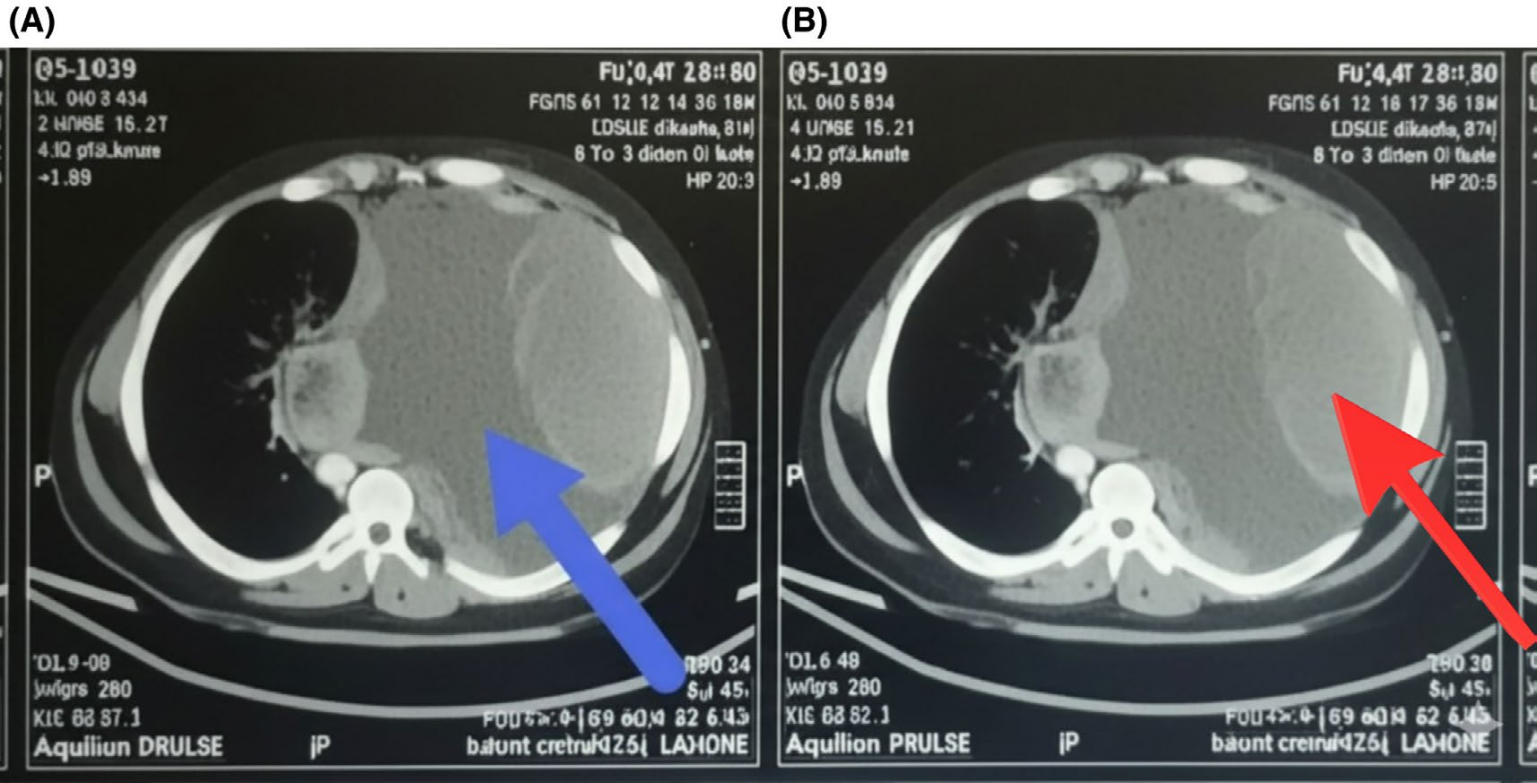
CT scan of the chest showing the soft tissue mass originating from the left rib and pleural fluid. (A) The blue arrow shows pleural fluid. (B) The red arrow shows a soft tissue density lesion in the periphery of the lung.

In the second paragraph of Section 2.3, the text “For further evaluation, a chest CT was performed; it showed a soft tissue‐enhancing lesion originating from the left rib (blue arrow in Figure 1) extending into the pleural cavity, causing a right‐sided mediastinal shift. The CT scan also showed pleural effusion around the left lung (red arrow in Figure 1).” was incorrect.

It should read: “For further evaluation, a chest CT was performed; it showed a soft tissue‐enhancing lesion originating from the left rib (red arrow in Figure 1) extending into the pleural cavity, causing a right‐sided mediastinal shift. The CT scan also showed pleural effusion around the left lung (blue arrow in Figure 1).”

Figure 1, along with its corrected caption, is provided below.

This correction does not alter the clinical interpretation, patient outcomes, or overall scientific conclusions of the case report.

We apologize for these errors.

